# Cumulative blood pressure load and cognitive decline in older adults: An observational analysis of two large cohorts

**DOI:** 10.1016/j.cccb.2024.100375

**Published:** 2024-12-16

**Authors:** Ying Xu, G. Peggy McFall, Lina Rydén, Johan Skoog, Edward Chang, Lucette A. Cysique, Katie Harris, Sarah Kedwell, Mei Ling Lim, Kaarin J. Anstey, Craig S. Anderson, Roger A. Dixon, Ingmar Skoog, Phillip J. Tully, Ruth Peters

**Affiliations:** a The George Institute for Global Health, Sydney, Australia; b Centre for Health Systems and Safety Research, Australian Institute of Health Innovation, Macquarie University, Sydney, Australia; c Department of Psychology (Science), University of Alberta, Alberta, Canada; d Department of Psychiatry and Neurochemistry, Institute of Neuroscience and Physiology, Sahlgrenska Academy, Centre for Ageing and Health (AgeCap) at the University of Gothenburg, Gothenburg, Sweden; e Region Västra Götaland, Sahlgrenska University Hospital, Neuropsychiatry Clinic, Gothenburg, Sweden; f University of Sydney Medical School, The University of Sydney, Sydney, Australia; g The Kirby Institute, Sydney, Australia; h School of Psychology, University of New South Wales, Sydney, Australia; i School of Biomedical Sciences, University of New South Wales, Sydney, Australia; j School of Population Health, University of New South Wales, Sydney, Australia; k Neuroscience Research Australia, Sydney, Australia; l Ageing Futures Institute, University of New South Wales, Sydney, Australia; m Institute of Science and Technology for Brain-inspired Intelligence, Fudan University, Shanghai, China; n Neuroscience and Mental Health Institute, University of Alberta, Alberta, Canada; o University of New England, Armidale, Australia

**Keywords:** Blood pressure, Cognitive decline, Cohort, Old adults

## Abstract

**Introduction:**

Cumulative blood pressure metrics may provide greater precision for measuring temporal risk exposure, especially in later life where data are mixed regarding associations of high blood pressure (BP) on cognitive function. We examined the relationship between greater cumulative exposure to high BP in later life and several domains of cognitive function.

**Methods:**

Individual cognitive assessment scores and BP measurements in older adults (age ≥70 years) at baseline and over approximately 8 years of follow-up were available in the population-based Canadian Victoria Longitudinal Study (VLS) and Swedish Gothenburg H70 Birth Cohort Studies (H70). Linear mixed models were used to quantify associations between cumulative systolic and diastolic BP and change in cognitive scores.

**Results:**

Each additional 100mmHg increase in cumulative BP was related to greater decline in the Rey Auditory Verbal Learning Test (RAVLT) List A, trials 1–5 total score over follow-up: -0.23 (95% confidence interval [CI] -0.32, -0.13) for systolic BP and -0.41 (95%CI -0.58, -0.23) for diastolic BP. Similarly increases cumulative systolic and diastolic BP were related to greater declines Digit Symbol Substitution Task (DSS) scores: -0.59 (95%CI -0.80, -0.38) and -1.04 (95% CI -1.40, -0.67), respectively. There were no associations of cumulative BP and temporal changes in general cognition, other measures of verbal episodic memory, or semantic fluency.

**Conclusions:**

Higher cumulative BP is associated with greater declines in RAVLT measured immediate memory span and complex attention, information processing speed and visuospatial scanning in older adults, but the scale of change is small. Additional research is required to further define these relationships and identify opportunities for prevention.

## Highlights

• Relationships between cumulative blood pressure and cognitive decline are understudied in late life.
• Data from two large cohort studies in two continents were analysed.
• Higher cumulative blood pressure was associated with greater declines in immediate memory span and complex attention, information processing speed and visuospatial scanning in older adults

## Introduction

A consistent relationship exists between higher blood pressure and increased risks of dementia and cognitive decline, but most of the evidence is derived from exposures in mid-life, whereas in later life, evidence has been more mixed [1,2]. One potential explanation is an inadequate measure of true risk-factor exposure over time, since unlike external risk factors such as smoking, blood pressure is physiological and present throughout life. Furthermore, systolic blood pressure typically rises with age in urbanised populations [3], such that proportionally more older adults will be exposed to higher blood pressures including values that are above current clinical thresholds for treatment. Resting office blood pressure measures that are taken at single or infrequent time points may therefore be a less useful exposure measure in older people [4].

Recent attention has been given to exploring the impact of cumulative blood pressure exposure in younger adults using methods analogous to calculating ‘pack-years’ for smoking. For example, summing blood pressure readings from repeated waves that are weighted by time intervals can allow an examination of the impact of cumulative exposure on both cognition [[5], [6], [7], [8], [9], [10]] and pathology [[11], [12], [13]]. Thus far, the evidence is strongest for cumulative exposure to systolic blood pressure in early adult life (∼20–40 years of age) and in midlife (∼40–60) to early later life (∼60–70) where higher levels of cumulative exposure are associated with an increased risk of incident dementia [5,7], and a decline in global cognition [[6], [7], [8]], executive function [6] and processing speed [14]. However, no relevant data are available for older people.

More information is required on the relationship of cumulative blood pressure and cognition in the older population, especially in those aged ≥70 years, who have the highest risk of cognitive decline and dementia. We aimed to determine associations between cumulative blood pressure exposure and several domains of cognitive function among older adults. We collected data from two cohort studies and adopted the same analyses.

## Methods

### Study design

Individual participant data were obtained from two population-based cohort studies with longitudinal measures of blood pressure and cognition in older adults: the Victoria Longitudinal Study (VLS) [[15], [16], [17], [18], [19], [20], [21]] and the Gothenburg H70 Birth Cohort Studies (H70) [[22], [23], [24]]. The studies represent populations of North America and Northern Europe, respectively. Both studies included participants aged ≥70 years who had measures of systolic and diastolic blood pressure and cognition during follow-up every 4 to 5 years. We collected blood pressure and cognitive function measures and similar covariates from the two studies.

### Measures of blood pressure and cognitive function

The H70 studies used the Swedish Population Register of birth dates to identify and approach a representative sample of the Gothenburg residential population aged 70 years [22,23]. Participants were assessed at baseline and at approximately 4–5 yearly intervals thereafter. The current investigation focused on the cohort born in 1930 who had their first examination in 2000 to 2002 (age 70 to 72 years), and followed up in 2005 to 2007 (aged 75 to 77 years) and 2009 to 2011 (aged 79 to 81 years) [23]. Single readings of systolic and diastolic blood pressure were available, measured at rest when seated using manual devices [24].

Cognitive function was assessed by trained research staff using a Swedish version of the Mini-Mental State Examination (MMSE) [24], a common cognitive screening tool, supplemented with tests of immediate and delayed word recall from the Alzheimer's Disease Assessment Scale-Cognitive (ADAS-Cog) and of semantic verbal fluency task (animals) [24].

The VLS study recruited adults aged 55–85 years from the community of Victoria, Canada, using community notices and advertisements between 1998 and 2002 [15]. Participants were assessed at baseline and at approximately 4 yearly intervals thereafter. Systolic and diastolic blood pressure were measured at rest whilst seated using digital devices, with 8 measures taken across 4 occasions within each wave of data collection, the mean pressure calculated from all measures were used for this study.

Cognitive tests included in the current study are the MMSE, the Rey Auditory Verbal Learning Test (RAVLT), total correct for immediate memory span (List A, trials 1–5), free recall (List B), and delayed recall (List A, trial 6) to assess verbal episodic memory [17,19,25], and the Wechsler Adult Intelligence Scale-Revised (WAIS-R) Digit Symbol Substitution (DSS) task to assess complex attention, information processing speed and visuospatial scanning (total correct responses in 90 seconds) [18,26]. To ensure these analyses were undertaken in a later life population, only VLS participants age ≥70 years at baseline were selected.

Cumulative blood pressure exposure was calculated for both systolic and diastolic blood pressure for each study using a technique analogous to that used to calculate ‘pack-years’ in smoking exposure and consistent with prior work in this area [27,28]. It conservatively assumed blood pressure remained the same until a subsequent wave recorded a different blood pressure level. The blood pressure at each wave was then multiplied by the years between that assessment and the next wave. The cumulative total exposure was obtained by summing the totals across the waves in each individual. For example, an individual with a baseline systolic blood pressure of 120 and subsequent readings of 130 after 4 years and 150 after 8 years would have a cumulative exposure of (120 × 4) + (130 × 4) + 150. The same principle was applied where blood pressure readings were available for waves before and after a missing reading. Blood pressure values were reported per 100mmHg to reduce the number of decimal places and to maximise clinical relevance for the results.

### Statistics

Change in cognitive function was calculated by subtracting the baseline score from the follow-up score such that a negative score indicated a decrease in cognitive performance. Because delayed recall was only available from wave two for H70, cumulative blood pressure exposure and cognitive change were recalculated to take account of this for all analyses related to the delayed recall outcome for H70. Analyses were run separately for systolic and diastolic blood pressure due to co-linearity.

Continuous variables with approximately symmetrical distribution were presented as mean with standard deviation. Both mean with standard deviation and median with interquartile interval were presented for the MMSE. Categorical variables were presented as number with percentage. The two cohorts were analysed separately using linear mixed models to examine the relationship between cumulative blood pressure and change in each cognitive score over the same period. Participants from two cohorts were combined to examine associations between blood pressure and change in MMSE. To be included in the analyses, a participant needed to have at least two measures of blood pressure. The longest follow-up was selected for each participant to calculate cumulative exposure to blood pressure, with two ends being the “first” (wave 2 for the delayed recall in H70, and baseline for the remaining cognitive measures) and the “last”. Participant needed to have the corresponding cognitive measures at these two waves of blood pressure measurements.

In the main analysis, models were fit adjusted for variables known to have an impact on blood pressure and cognition [29,30]. These were age, sex, current smoking, education, presence of diabetes mellitus, treatment with antihypertensive medications at baseline (VLS, H70) or antihypertensive use during follow-up (available for H70 only), and the corresponding baseline cognitive measure. Trajectory of systolic blood pressure over follow-up was also included to take account of either consistently rising, or consistently falling blood pressure or neither. In adjusted analyses, participants with missing information for any of these variables were excluded. Unadjusted analysis was also conducted. As sex-specific patterns have been reported for dementia, cognitive decline and their risk factors [31], all analyses were run separately by reported sex (male/female). Sensitivity analyses were undertaken for cumulative mean systolic blood pressures >100 mmHg at each wave (i.e. using the same methodology but for a blood pressure of 120, a value of 20 was used) and similarly for pressures >120 mmHg and for cohorts within each study where participants had attended study visits at all waves, to examine the potential impact of loss to follow-up or withdrawal. Additionally, quadratic blood pressure terms were assessed for non-linear relationships. Finally, due to the relationship between dementia or developing dementia and falling blood pressure [32], we excluded those who had dementia recorded at baseline or during follow-up waves (present in H70 only) and those with a baseline MMSE score <24 or an MMSE score that fell to ≤21 during follow up (VLS and H70). Statistical significance was set at *p*<0.01 to account for multiple comparisons. All analyses were undertaken with Stata MP 18 (StataCorp LP, College Station, TX).

## Results

There were 604 H70 participants and 557 VLS participants included in the analyses (Table 1, Figures S1 and S2). Follow-up was 8.5 ± 2.0 (mean ± standard deviation) years in the H70 and 8.5 ± 4.0 years in the VLS.

**Table 1:** Characteristics of the analytical samples.

Sample characteristics Mean ± SD unless otherwise specified	Gothenburg H70 Birth Cohort Studies (*n*=604)	Victoria Longitudinal Study on Aging (*n*=557)
Age (years)	70.6 ± 0.3	77.6 ± 4.9
Female, n (%)	361 (59.8)	342 (61.4)
Blood pressure (mmHg)		
Systolic baseline (wave 1)	154.9 ± 22.2	131.6 ± 15.1
Diastolic baseline (wave 1)	83.8 ± 10.6	74.8 ± 9.4
Systolic wave 2	149.4 ± 20.9	129.9 ± 15.4
Diastolic wave 2	80.8 ± 9.7	71.0 ± 9.7
Systolic wave 3	137.5 ± 18.6	129.9 ± 15.7
Diastolic wave 3	73.6 ± 10.3	68.7 ± 9.0
Systolic wave 4	–	130.0 ± 16.2
Diastolic wave 4	–	69.7 ± 8.9
Antihypertensives at baseline (VLS) or anytime (H70), n (%)	504 (83.4)	151 (27.1)
Diabetes mellitus, n (%)	48 (8.0)	41 (7.5)
Mini-Mental State Examination	27.9 ± 2.6	28.4 ± 1.4
Mini-Mental State Examination, median (IQR)	28 (27–29)	29 (28–29)
Current smoker, n (%)	92 (15.6)	34 (6.2)
Body mass index, kg/m^2^	27.1 ± 4.3	26.5 ± 4.3
Education, n (%) in H70	327 (54.8) (compulsory school)	14.8 ± 3.0 (years of education)
	188 (31.5) (more than compulsory but no university)	
	82 (13.7) (university)	
Follow up (years)	8.5 ± 2.0 (*n*=495)	8.5 ± 4.0 (*n*=388)

IQR denotes interquartile range, SD standard deviation. Table 2 Cumulative exposure to blood pressure and change in cognition VLS

In the VLS (Table 2), each additional 100mmHg exposure to systolic blood pressure during follow-up was associated with a greater decline in total scores for RAVLT List A, trials 1–5 of -0.23 (95% Confidence Intervals (-0.32, -0.13). The commensurate value for diastolic blood pressure was a greater decline of -0.41 (-0.58, -0.23). Greater declines in WAIS-R DSS score of -0.59 (-0.80, -0.38) and -1.04 (-1.40, -0.67) were also observed for each additional 100mmHg exposure to systolic and diastolic blood pressure, respectively, during follow-up.

**Table 2:** cumulative exposure to blood pressure and change in cognition VLS.

Cognitive tests	Blood pressure	*n*	β (95% confidence interval)	*p*
Immediate memory span (RAVLT List A, trials 1–5)	Systolic	327	-0.23 (-0.32, -0.13)	<0.0001
	Diastolic		-0.41 (-0.58, -0.23)	<0.0001
Free recall (RAVLT Interference List B)	Systolic	108	-0.01 (-0.09, 0.07)	0.78
	Diastolic		-0.004 (-0.16, 0.15)	0.96
Delayed recall (RAVLT List A, trial 6)	Systolic	108	-0.03 (-0.17, 0.12)	0.74
	Diastolic		-0.02 (-0.30, 0.25)	0.86
Complex attention, information processing speed, and visuospatial scanning (WAIS-R DSS)	Systolic	247	-0.59 (-0.80, -0.38)	<0.0001
	Diastolic		-1.04 (-1.40, -0.67)	<0.0001
General cognition (MMSE)	Systolic	358	-0.04 (-0.09, 0.01)	0.09
	Diastolic		-0.09 (-0.17, -0.01)	0.03

DSS denotes Digit Symbol Substitution task, MMSE Mini Mental State Examination, RAVLT Rey Auditory Verbal Learning Test, VLS Victoria Longitudinal Study, WAIS-R Wechsler Adult Intelligence Scale-Revised.

Blood pressure represents cumulative blood pressure (per 100mmHg higher). Linear mixed models were used, adjusting for age, sex, current smoking, education, body mass index, presence of diabetes, treatment with antihypertensive medications at baseline, trajectory of systolic blood pressure change, and the corresponding baseline measure of cognitive function. Negative relationship indicates that greater cumulative blood pressure exposure is associated with lower final cognitive scores in cross-temporal cumulative analyses and with greater fall in cognitive score over time in longitudinal cumulative analyses.

There were no associations between cumulative blood pressure over follow-up and changes in general cognition (MMSE) or RAVLT (List B or List A trial 6) or ADAS-Cog assessed memory over the same period (Tables 2,3). Similarly, there were no relationships between cumulative pressure and semantic fluency (drawing on executive function, semantic abilities, and language) (available for H70 only). When VLS and H70 participants were combined (n=796), per 100mmHg higher cumulative blood pressures were not related to changes in MMSE, -0.03 (-0.08, 0.02) for systolic and -0.06 (-0.15, 0.03) for diastolic. Conducting analyses by sex found similar patterns as in the main analyses (Tables S1–4). Yet, the relationship between higher cumulative diastolic blood pressure and greater declines in MMSE and in semantic fluency were observed in female VLS and H70 participants, respectively.

**Table 3:** cumulative exposure to blood pressure and change in cognition H70.

Cognitive tests	Blood pressure	*n*	β (95% confidence interval)	*p*
Immediate recall	Systolic	445	0.07 (-0.0003, 0.13)	0.05
	Diastolic		0.09 (-0.04, 0.22)	0.18
Delayed recall (from 2005)	Systolic	304	-0.13 (-0.36, 0.09)	0.25
	Diastolic		-0.02 (-0.51, 0.46)	0.93
Semantic fluency	Systolic	445	-0.16 (-0.31, 0.00)	0.05
	Diastolic		-0.33 (-0.62, -0.03)	0.03
General cognition (MMSE)	Systolic	438	-0.02 (-0.11, 0.07)	0.69
	Diastolic		0.01 (-0.17, 0.19)	0.91

MMSE denotes Mini Mental State Examination.

Blood pressure represents cumulative blood pressure (per 100mmHg higher). Linear mixed models were used, adjusting for age, sex, current smoking, education, body mass index, presence of diabetes, treatment with antihypertensive medications, trajectory of systolic blood pressure change, and the corresponding baseline measure of cognitive function. Negative relationship indicates that greater cumulative blood pressure exposure is associated with lower final cognitive scores in cross-temporal cumulative analyses and with greater fall in cognitive score over time in longitudinal cumulative analyses.

Sensitivity analysis of cohorts assessing the impact of cumulative systolic blood pressures greater than 100mmHg and greater than 120mmHg showed the same results as in the main analyses. Analysis of cohorts where participants had attended all waves showed a similar pattern of results but without statistical significance in RAVLT List A, trials 1–5 and an additional relationship between higher cumulative diastolic blood pressure and greater decline in MMSE in VLS participants (Tables S5,6). In unadjusted analyses, we additionally found relationships between higher cumulative blood pressures and greater decline in MMSE in VLS participants and greater decline in semantic fluency in H70 participants compared to the main analyses (Tables S7,8). There were no quadratic relationships. Excluding VLS participants who had a baseline MMSE score <24 or an MMSE score that fell to ≤21 during follow up did not change the results (Table S9). Yet, when these participants were excluded along with those who had dementia recorded at baseline or during follow-up waves (present in H70 only), we observed associations between higher cumulative blood pressures and lower declines in the MMSE (Table S10).

## Discussion

Analyses of two later life longitudinal population-based cohort studies found greater cumulative exposure to both systolic and diastolic blood pressure to be associated with greater declines in performance on the immediate memory span (RAVLT List A, trials 1–5) and the WAIS-R DSS (a task assessing complex attention, information processing speed and visuospatial scanning) over an average of 8.5 years follow-up. Yet, RAVLT and DSS were available only in the VLS and the estimated greater changes in raw scores are small. In female participants, we observed relationships between cumulative diastolic blood pressure exposure and general cognition assessed using a brief screening tool (MMSE) and semantic fluency. Discrepancies in identified relationships between unadjusted and adjusted analyses suggested the role of confounding factors and attrition due to missing values in covariates.

This fits with prior work supporting the associations between complex attention, information processing speed, cardiovascular risk factors and cardiovascular diseases [12,14,33]. The Atherosclerosis Risk In Communities Study (ARIC) found a greater decline in attention and information processing speed also measured using a DSS task over a 20-year period (median follow-up 19.1 years) in those with hypertension or prehypertension compared to normotensive counterparts, albeit in a younger population (mean age at baseline ∼58 years) [14]. Interestingly, unlike other studies [6], the association between blood pressure and semantic fluency we found only presented in females and was only for diastolic blood pressure, although the executive function domain, tapped into by the semantic fluency task, is often reported as being one of the cognitive domains most impacted by vascular risk factors such as blood pressure [34].

Our findings have real-world relevance since people use memory to retain new knowledge and changes in abilities reflecting high-order cognition can impact high-level day-to-day function and decision-making [35]. In fact, multiple domains of cognition have been found to be related to severity of hypertension, including language, processing speed, visuospatial abilities, and memory [36]. Thus, identifying older adults who may be at risk of cognitive decline by assessing cognitive domains carefully, and at an early stage is important to allow timely treatment to be put in place. Furthermore, understanding the potential role of cumulative blood pressure raises the possibility of reducing risk by decreasing exposure to higher blood pressures, with recent work further reinforcing the potential for blood pressure lowering to reduce incident dementia [37,38] and cognitive impairment [39]. Finally, since blood pressure falls around 5 years before a dementia diagnosis [32], associations between higher blood pressures and greater declines in cognitive functions were expected to become stronger after removing those who had any possible cognitive impairments. Yet, we did not observe this, and conversely, inverse associations were found for the MMSE in the H70. The inverse associations, however, were likely not clinically relevant, as a reliable change at 1.5-year intervals would need a change in raw scores of at least 2 to 4 points [40].

Uncertainties remain, however, and data are still mixed regarding the impact of blood pressure lowering on specific cognitive domains [41] and on the ideal blood pressure levels for the protection of brain health in older adults [38]. Our work highlights the potential for long-term blood pressure lowering, reducing cumulative exposure to protect decline in immediate memory span and complex attention in this fast-growing older adult population, and the need for additional work in this area.

Strengths of our study include the use of established population-based cohort studies with later life populations and similar lengths of follow-up, which provide a unique opportunity to examine similar data from two different continents on a range of cognitive domains. However, there remain limitations to our work. Whilst it was possible to largely standardise covariates across the two studies and the available data allowed adjustments for the key factors known to influence cognition, it remains possible that unmeasured confounding may have influenced the results, including the presence of non-vascular brain pathology and genetic risk factors relevant to cognition. The VLS and H70 studies also have different designs, and although they used established cognitive measures, these differed by study with unequal psychometric properties in their ability to measure cognitive change, meaning that an exact like-for-like comparison was not possible. Furthermore, the complex attention and information processing speed tests representing high order cognitive abilities had optimal psychometric properties for measuring cognitive change over time with an infinite number of scores [42]. Scores of the RAVLT immediate memory span (List A, trials 1–5) have a wide range (0 to 75). These contrasted with the other tasks which have a restricted range of possible scores (verbal memory, MMSE) with consequent reduction in ability to measure subtle change, greater susceptibility to ceiling effects and which are more influenced by vocabulary and education (MMSE, semantic fluency) [42]. We also selected the longest follow-up time for each individual to calculate cumulative exposure and cognitive change and this precluded the use of more sophisticated reliable change measures due to variations in time gaps between the two cognitive measures [43]. The cognitive change represents the difference between two longitudinal cognitive measures, however, individual variations are likely in pathways of changes [17]. Additionally, while the approximately 4-year intervals between collections of blood pressure data are not unusual in cohort studies, it necessarily restricts the granularity available for the calculation of cumulative exposure. Longitudinal blood pressures were equally weighted, as there is no evidence to suggest they are valued differently with ageing after 70 years old. The study populations also varied, for example, different blood pressure levels at baseline potentially indicating one of the challenges in interpreting data from diverse cohorts and time periods where unique case-mix and blood pressure treatment pathways may apply. Nevertheless, our results agree with prior work and highlight a need for an additional focus on exposure to higher blood pressure over time and the potential for additional blood pressure control in later life.

## Relevance for future research

We need more research adopting cumulative blood pressure measurements with closer intervals over long follow-ups and standardised collection on information for covariates with limited missing values. Cognitive test batteries covering a broad range of cognitive domains and sex difference should also be considered. Evaluation of this area is timely since we have an ageing population who are at greater risk of higher cumulative exposure than at younger ages and widely available blood pressure lowering treatments that may bring cognitive benefit. There are multiple mechanisms, e.g., reduced brain perfusion and cortical thickness [44], by which high blood pressure may influence cognition and work in these areas is important. The next steps also include a need to understand whether reducing cumulative exposure and by how much would help protect cognition in this age group.

## Conclusions

Greater cumulative exposure to higher blood pressure over ∼8 years in later life was associated with a greater decline in immediate memory span and complex attention and information processing speed over the same period in the Canadian cohort. Since memory, complex attention and information processing speed are crucial to learning and maintaining high-level day-to-day abilities such as road safety, it is important to further evaluate the role of blood pressure reduction and cognition to understand whether this may provide protection for critical cognitive domains in this fast-growing age group.

## Disclosures

Kaarin J Anstey declares a speaker fee from Roche (2023). The other authors have no competing interests to declare.

## Sources of funding

This work was supported by the National Health and Medical Research Council (NHMRC) of Australia project (APP1160373).

Kaarin J Anstey is funded by ARC Laureate Fellowship FL190100011.

Roger A Dixon's contribution is supported by the Canadian Consortium on Neurodegeneration in Aging and funded by a partnership of Alberta Innovates (#G2020000063) and the Canadian Institutes of Health Research (#163902). The VLS is funded by the US National Institute on Aging (R01 #AG008235).

H70 studies were supported by grants from the Swedish state under the agreement between the Swedish government and the county councils, the ALF-agreement (ALF965812, ALF 716681). Swedish Research Council (2019-01096, 2022-00882, 2017-00639), Swedish Research Council for Health, Working Life and Wellfare (2013-1202, 2018-00471, 2013-2300, 2013-2496), Konung Gustaf V:s och Drottning Victorias Frimurarestiftelse, Hjärnfonden (FO2018-0214, FO2019-0163, FO2020-0235,), Alzheimerfonden (AF-940139, AF-968441, AF-980935), Eivind och Elsa K:son Sylvans stiftelse, The Alzheimer's Association (ZEN-01-3151, IIRG-00-2159).

## CRediT authorship contribution statement

**Ying Xu:** Conceptualization, Data curation, Formal analysis, Investigation, Methodology, Project administration, Resources, Supervision, Validation, Writing – original draft, Writing – review & editing. **G. Peggy McFall:** Conceptualization, Data curation, Investigation, Methodology, Validation, Writing – review & editing. **Lina Rydén:** Data curation, Investigation, Writing – review & editing. **Johan Skoog:** Data curation, Investigation, Writing – review & editing. **Edward Chang:** Data curation, Investigation, Validation, Writing – review & editing. **Lucette A. Cysique:** Investigation, Writing – review & editing. **Katie Harris:** Investigation, Methodology, Validation, Writing – review & editing. **Sarah Kedwell:** Investigation, Validation, Writing – review & editing. **Mei Ling Lim:** Investigation, Validation, Writing – review & editing. **Kaarin J. Anstey:** Data curation, Funding acquisition, Investigation, Writing – review & editing. **Craig S. Anderson:** Funding acquisition, Investigation, Writing – review & editing. **Roger A. Dixon:** Data curation, Funding acquisition, Investigation, Validation, Writing – review & editing. **Ingmar Skoog:** Data curation, Funding acquisition, Investigation, Writing – review & editing. **Phillip J. Tully:** Conceptualization, Data curation, Funding acquisition, Investigation, Writing – review & editing. **Ruth Peters:** Conceptualization, Data curation, Formal analysis, Funding acquisition, Investigation, Methodology, Project administration, Resources, Supervision, Writing – original draft, Writing – review & editing.

## Declaration of competing interest

The authors declare the following financial interests/personal relationships which may be considered as potential competing interests: Kaarin J Anstey declares a speaker fee from Roche (2023). The other authors have no competing interests to declare.

## Appendix Supplementary materials

Image, application 1
