# Supplementary material for: Cumulative blood pressure load and cognitive decline in older adults: An observational analysis of two large cohorts
Source: Cereb Circ Cogn Behav. 2024 Dec 16;8:100375. doi: 10.1016/j.cccb.2024.100375 (PMC11730254; doi:10.1016/j.cccb.2024.100375)
Supplement: Supplementary file 1 [file mmc1.docx]

This supplementary has been provided by the authors to give readers additional information about their work.

Supplement to:

Cumulative blood pressure load and cognitive decline in older adults: an observational analysis of two large cohorts.

Contents

[Figure S1 Flow chart of analysed VLS participants 1](#_Toc183598069)

[Figure S2 Flow chart of analysed H70 participants 2](#_Toc183598070)

[Table S1 cumulative exposure to blood pressure and change in cognition VLS, Male 3](#_Toc183598071)

[Table S2 cumulative exposure to blood pressure and change in cognition VLS, Female 4](#_Toc183598072)

[Table S3 cumulative exposure to blood pressure and change in cognition H70, Male 5](#_Toc183598073)

[Table S4 cumulative exposure to blood pressure and change in cognition H70, Female 6](#_Toc183598074)

[Table S5 cumulative exposure to blood pressure and change in cognition VLS, cohort analyses 7](#_Toc183598075)

[Table S6 cumulative exposure to blood pressure and change in cognition H70, cohort analyses 8](#_Toc183598076)

[Table S7 cumulative exposure to blood pressure and change in cognition VLS, unadjusted analyses 9](#_Toc183598077)

[Table S8 cumulative exposure to blood pressure and change in cognition H70, unadjusted analyses 10](#_Toc183598078)

[Table S9 cumulative exposure to blood pressure and change in cognition VLS, excluding those with possible dementia 11](#_Toc183598079)

[Table S10 cumulative exposure to blood pressure and change in cognition H70, excluding those with possible dementia 12](#_Toc183598080)


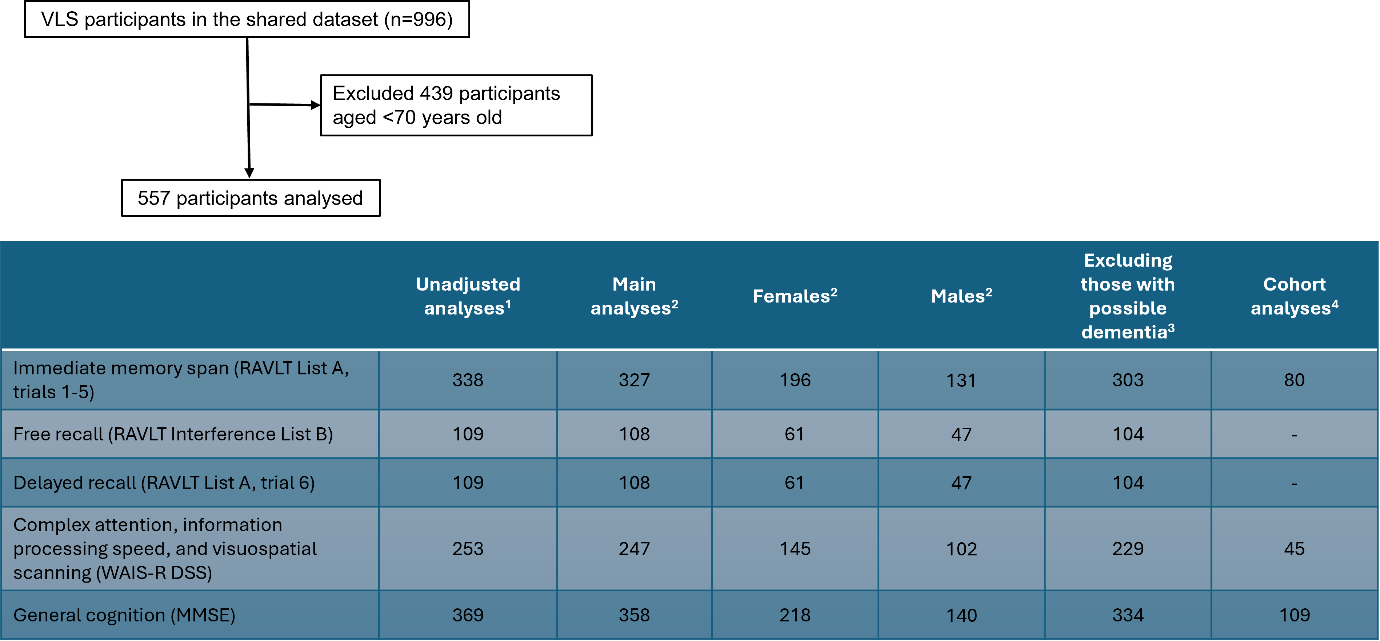


# Figure S1 Flow chart of analysed VLS participants

DSS denotes Digit Symbol Substitution task, MMSE Mini Mental State Examination, RAVLT Rey Auditory Verbal Learning Test, VLS Victoria Longitudinal Study, WAIS-R Wechsler Adult Intelligence Scale-Revised.

^1^Depending on availability of the baseline and the “final” cognitive measures.

^2^Excluding those who had missing values for any of the covariates compared to the unadjusted analyses.

^3^Excluding those with a baseline MMSE score <24 or an MMSE score that fell to ≤21 during follow up.

^4^Only including participants who had attended study visits at all waves.


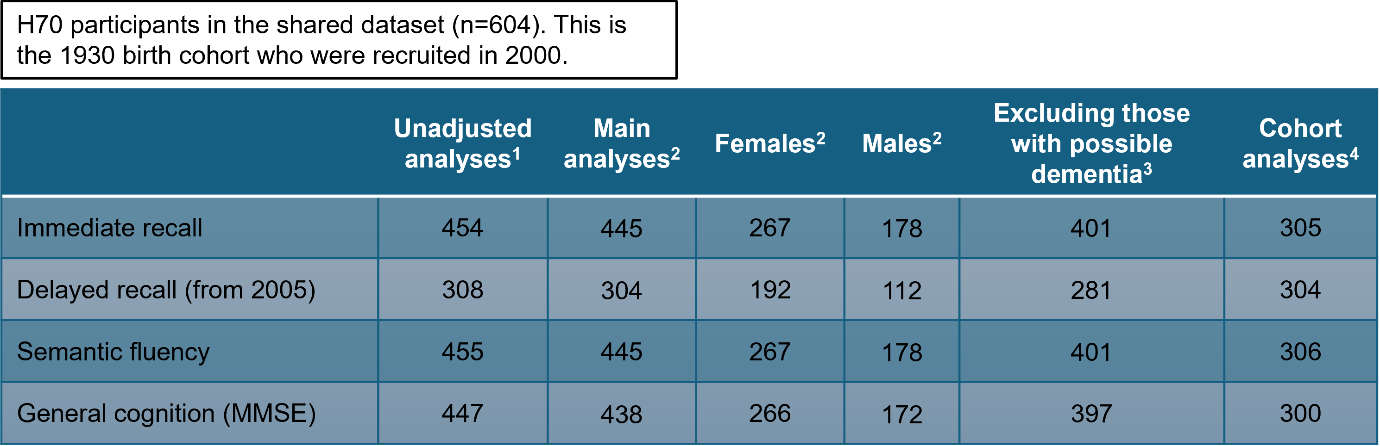


# Figure S2 Flow chart of analysed H70 participants

MMSE denotes Mini Mental State Examination.

^1^Depending on availability of the baseline and the “final” cognitive measures.

^2^Excluding those who had missing values for any of the covariates compared to the unadjusted analyses.

^3^Excluding those with a baseline MMSE score <24 or an MMSE score that fell to ≤21 during follow up, and those who had dementia recorded at baseline or during follow-up waves.

^4^Only including participants who had attended study visits at all waves.

# Table S1 cumulative exposure to blood pressure and change in cognition VLS, Male

| Cognitive tests | Blood pressure | *n* | β (95% confidence interval) | *p* |
| --- | --- | --- | --- | --- |
| Immediate memory span (RAVLT List A, trials 1-5) | Systolic | 131 | -0.26 (-0.39, -0.12) | 0.0002 |
|  | Diastolic |  | -0.38 (-0.62, -0.14) | 0.002 |
| Free recall (RAVLT Interference List B) | Systolic | 47 | 0.01 (-0.12, 0.13) | 0.90 |
|  | Diastolic |  | 0.03 (-0.19, 0.26) | 0.76 |
| Delayed recall (RAVLT List A, trial 6) | Systolic | 47 | 0.08 (-0.14, 0.31) | 0.46 |
|  | Diastolic |  | 0.15 (-0.25, 0.54) | 0.47 |
| Complex attention, information processing speed, and visuospatial scanning (WAIS-R) | Systolic | 102 | -0.48 (-0.79, -0.17) | 0.002 |
|  | Diastolic |  | -0.71 (-1.25, -0.17) | 0.01 |
| General cognition (MMSE) | Systolic | 140 | -0.01 (-0.08, 0.06) | 0.79 |
|  | Diastolic |  | -0.02 (-0.15, 0.12) | 0.82 |

DSS denotes Digit Symbol Substitution task, MMSE Mini Mental State Examination, RAVLT Rey Auditory Verbal Learning Test, VLS Victoria Longitudinal Study, WAIS-R Wechsler Adult Intelligence Scale-Revised.

Blood pressure represents cumulative blood pressure (per 100mmHg higher). Linear mixed models were used, adjusting for age, current smoking, education, body mass index, presence of diabetes, treatment with antihypertensive medications at baseline, trajectory of systolic blood pressure change, and the corresponding baseline measure of cognitive function.

Negative relationships indicate that greater cumulative blood pressure exposures are associated with greater declines in cognitive scores over time.

# Table S2 cumulative exposure to blood pressure and change in cognition VLS, Female

| Cognitive tests | Blood pressure | *n* | β (95% confidence interval) | *p* |
| --- | --- | --- | --- | --- |
| Immediate memory span (RAVLT List A, trials 1-5) | Systolic | 181 | -0.20 (-0.34, -0.06) | 0.006 |
|  | Diastolic |  | -0.42 (-0.67, -0.17) | 0.001 |
| Free recall (RAVLT Interference List B) | Systolic | 59 | 0.05 (-0.07, 0.17) | 0.42 |
|  | Diastolic |  | 0.09 (-0.14, 0.32) | 0.45 |
| Delayed recall (RAVLT List A, trial 6) | Systolic | 59 | -0.07 (-0.27, 0.13) | 0.49 |
|  | Diastolic |  | -0.14 (-0.52, 0.25) | 0.49 |
| Complex attention, information processing speed, and visuospatial scanning (WAIS-R DSS) | Systolic | 132 | -0.62 (-0.91, -0.34) | <0.0001 |
|  | Diastolic |  | -1.21 (-1.72, -0.71) | <0.0001 |
| General cognition (MMSE) | Systolic | 200 | -0.06 (-0.12, 0.002) | 0.06 |
|  | Diastolic |  | -0.14 (-0.24, -0.03) | 0.01 |

DSS denotes Digit Symbol Substitution task, MMSE Mini Mental State Examination, RAVLT Rey Auditory Verbal Learning Test, VLS Victoria Longitudinal Study, WAIS-R Wechsler Adult Intelligence Scale-Revised.

Blood pressure represents cumulative blood pressure (per 100mmHg higher). Linear mixed models were used, adjusting for age, current smoking, education, body mass index, presence of diabetes, treatment with antihypertensive medications at baseline, trajectory of systolic blood pressure change, and the corresponding baseline measure of cognitive function.

Negative relationships indicate that greater cumulative blood pressure exposures are associated with greater declines in cognitive scores over time.

# Table S3 cumulative exposure to blood pressure and change in cognition H70, Male

| Cognitive tests | Blood pressure | *n* | β (95% confidence interval) | *p* |
| --- | --- | --- | --- | --- |
| Immediate recall | Systolic | 178 | 0.04 (-0.06, 0.15) | 0.43 |
|  | Diastolic |  | 0.07 (-0.14, 0.27) | 0.53 |
| Delayed recall (from 2005) | Systolic | 112 | -0.39 (-0.78, -0.01) | 0.04 |
|  | Diastolic |  | -0.13 (-0.94, 0.68) | 0.76 |
| Semantic fluency | Systolic | 178 | -0.10 (-0.35, 0.15) | 0.45 |
|  | Diastolic |  | -0.14 (-0.61, 0.33) | 0.55 |
| General cognition (MMSE) | Systolic | 172 | -0.09 (-0.22, 0.04) | 0.18 |
|  | Diastolic |  | -0.11 (-0.35, 0.14) | 0.39 |

MMSE denotes Mini Mental State Examination.

Blood pressure represents cumulative blood pressure (per 100mmHg higher). Linear mixed models were used, adjusting for age, current smoking, education, body mass index, presence of diabetes, treatment with antihypertensive medications, trajectory of systolic blood pressure change, and the corresponding baseline measure of cognitive function.

Negative relationships indicate that greater cumulative blood pressure exposures are associated with greater declines in cognitive scores over time.

# Table S4 cumulative exposure to blood pressure and change in cognition H70, Female

| Cognitive tests | Blood pressure | *n* | β (95% confidence interval) | *p* |
| --- | --- | --- | --- | --- |
| Immediate recall | Systolic | 267 | 0.07 (-0.01, 0.16) | 0.09 |
|  | Diastolic |  | 0.09 (-0.07, 0.26) | 0.26 |
| Delayed recall (from 2005) | Systolic | 192 | -0.07 (-0.35, 0.22) | 0.64 |
|  | Diastolic |  | -0.05 (-0.66, 0.56) | 0.87 |
| Semantic fluency | Systolic | 267 | -0.22 (-0.42, -0.02) | 0.03 |
|  | Diastolic |  | -0.51 (-0.91, -0.12) | 0.01 |
| General cognition (MMSE) | Systolic | 266 | 0.02 (-0.11, 0.15) | 0.82 |
|  | Diastolic |  | 0.08 (-0.17, 0.33) | 0.54 |

MMSE denotes Mini Mental State Examination.

Blood pressure represents cumulative blood pressure (per 100mmHg higher). Linear mixed models were used, adjusting for age, current smoking, education, body mass index, presence of diabetes, treatment with antihypertensive medications, trajectory of systolic blood pressure change, and the corresponding baseline measure of cognitive function.

Negative relationships indicate that greater cumulative blood pressure exposures are associated with greater declines in cognitive scores over time.

# Table S5 cumulative exposure to blood pressure and change in cognition VLS, cohort analyses

| Cognitive tests | Blood pressure | *n* | β (95% confidence interval) | *p* |
| --- | --- | --- | --- | --- |
| Immediate memory span (RAVLT List A, trials 1-5) | Systolic | 80 | -0.14 (-0.72, 0.43) | 0.62 |
|  | Diastolic |  | -0.62 (-1.56, 0.31) | 0.19 |
| Free recall (RAVLT Interference List B) | Systolic | - | - | - |
|  | Diastolic |  | - | - |
| Delayed recall (RAVLT List A, trial 6) | Systolic | - | - | - |
|  | Diastolic |  | - | - |
| Complex attention, information processing speed, and visuospatial scanning (WAIS-R DSS) | Systolic | 45 | -1.96 (-3.50, -0.42) | 0.01 |
|  | Diastolic |  | -3.25 (-5.16, -1.33) | 0.001 |
| General cognition (MMSE) | Systolic | 109 | -0.11 (-0.32, 0.09) | 0.29 |
|  | Diastolic |  | -0.44 (-0.75, -0.12) | 0.007 |

DSS denotes Digit Symbol Substitution task, MMSE Mini Mental State Examination, RAVLT Rey Auditory Verbal Learning Test, VLS Victoria Longitudinal Study, WAIS-R Wechsler Adult Intelligence Scale-Revised.

Blood pressure represents cumulative blood pressure (per 100mmHg higher). Linear mixed models were used, adjusting for age, sex, current smoking, education, body mass index, presence of diabetes, treatment with antihypertensive medications at baseline, trajectory of systolic blood pressure change, and the corresponding baseline measure of cognitive function.

Negative relationships indicate that greater cumulative blood pressure exposures are associated with greater declines in cognitive scores over time.

# Table S6 cumulative exposure to blood pressure and change in cognition H70, cohort analyses

| Cognitive tests | Blood pressure | *n* | β (95% confidence interval) | *p* |
| --- | --- | --- | --- | --- |
| Immediate recall | Systolic | 305 | 0.03 (-0.11, 0.18) | 0.65 |
|  | Diastolic |  | -0.12 (-0.43, 0.20) | 0.47 |
| Delayed recall (from 2005) | Systolic | 304 | -0.13 (-0.36, 0.09) | 0.25 |
|  | Diastolic |  | -0.02 (-0.51, 0.46) | 0.93 |
| Semantic fluency | Systolic | 306 | 0.11 (-0.22, 0.45) | 0.50 |
|  | Diastolic |  | -0.03 (-0.76, 0.69) | 0.93 |
| General cognition (MMSE) | Systolic | 300 | 0.01 (-0.15, 0.18) | 0.86 |
|  | Diastolic |  | 0.10 (-0.26, 0.45) | 0.60 |

MMSE denotes Mini Mental State Examination.

Blood pressure represents cumulative blood pressure (per 100mmHg higher). Linear mixed models were used, adjusting for age, sex, current smoking, education, body mass index, presence of diabetes, treatment with antihypertensive medications, trajectory of systolic blood pressure change, and the corresponding baseline measure of cognitive function.

Negative relationships indicate that greater cumulative blood pressure exposures are associated with greater declines in cognitive scores over time.

# Table S7 cumulative exposure to blood pressure and change in cognition VLS, unadjusted analyses

| Cognitive tests | Blood pressure | *n* | β (95% confidence interval) | *p* |
| --- | --- | --- | --- | --- |
| Immediate memory span (RAVLT List A, trials 1-5) | Systolic | 338 | -0.25 (-0.34, -0.17) | <0.0001 |
|  | Diastolic |  | -0.46 (-0.61, -0.30) | <0.0001 |
| Free recall (RAVLT Interference List B) | Systolic | 109 | -0.07 (-0.17, 0.04) | 0.24 |
|  | Diastolic |  | -0.13 (-0.33, 0.06) | 0.18 |
| Delayed recall (RAVLT List A, trial 6) | Systolic | 109 | -0.08 (-0.22, 0.06) | 0.27 |
|  | Diastolic |  | -0.12 (-0.38, 0.13) | 0.34 |
| Complex attention, information processing speed, and visuospatial scanning (WAIS-R DSS) | Systolic | 253 | -0.58 (-0.77, -0.40) | <0.0001 |
|  | Diastolic |  | -1.05 (-1.37, -0.73) | <0.0001 |
| General cognition (MMSE) | Systolic | 369 | -0.05 (-0.10, -0.01) | 0.009 |
|  | Diastolic |  | -0.11 (-0.19, -0.04) | 0.002 |

DSS denotes Digit Symbol Substitution task, MMSE Mini Mental State Examination, RAVLT Rey Auditory Verbal Learning Test, VLS Victoria Longitudinal Study, WAIS-R Wechsler Adult Intelligence Scale-Revised.

Blood pressure represents cumulative blood pressure (per 100mmHg higher). Linear mixed models were used. Negative relationships indicate that greater cumulative blood pressure exposures are associated with greater declines in cognitive scores over time.

# Table S8 cumulative exposure to blood pressure and change in cognition H70, unadjusted analyses

| Cognitive tests | Blood pressure | *n* | β (95% confidence interval) | *p* |
| --- | --- | --- | --- | --- |
| Immediate recall | Systolic | 454 | 0.04 (-0.03, 0.11) | 0.27 |
|  | Diastolic |  | 0.05 (-0.09, 0.18) | 0.49 |
| Delayed recall (from 2005) | Systolic | 308 | -0.09 (-0.35, 0.16) | 0.47 |
|  | Diastolic |  | 0.04 (-0.50, 0.58) | 0.88 |
| Semantic fluency | Systolic | 455 | -0.24 (-0.40, -0.08) | 0.003 |
|  | Diastolic |  | -0.47 (-0.77, -0.18) | 0.002 |
| General cognition (MMSE) | Systolic | 447 | 0.04 (-0.05, 0.12) | 0.39 |
|  | Diastolic |  | 0.10 (-0.06, 0.26) | 0.21 |

MMSE denotes Mini Mental State Examination.

Blood pressure represents cumulative blood pressure (per 100mmHg higher). Linear mixed models were used. Negative relationships indicate that greater cumulative blood pressure exposures are associated with greater declines in cognitive scores over time.

# Table S9 cumulative exposure to blood pressure and change in cognition VLS, excluding those with possible dementia

| Cognitive tests | Blood pressure | *n* | β (95% confidence interval) | *p* |
| --- | --- | --- | --- | --- |
| Immediate memory span (RAVLT List A, trials 1-5) | Systolic | 303 | -0.19 (-0.28, -0.09) | 0.0002 |
|  | Diastolic |  | -0.33 (-0.50, -0.16) | 0.0002 |
| Free recall (RAVLT Interference List B) | Systolic | 104 | -0.02 (-0.10, 0.07) | 0.71 |
|  | Diastolic |  | -0.003 (-0.16, 0.15) | 0.96 |
| Delayed recall (RAVLT List A, trial 6) | Systolic | 104 | -0.02 (-0.17, 0.13) | 0.77 |
|  | Diastolic |  | -0.02 (-0.29, 0.26) | 0.90 |
| Complex attention, information processing speed, and visuospatial scanning (WAIS-R DSS) | Systolic | 229 | -0.57 (-0.78, -0.36) | <0.0001 |
|  | Diastolic |  | -0.98 (-1.36, -0.61) | <0.0001 |
| General cognition (MMSE) | Systolic | 334 | -0.005 (-0.04, 0.03) | 0.79 |
|  | Diastolic |  | -0.02 (-0.09, 0.04) | 0.46 |

DSS denotes Digit Symbol Substitution task, MMSE Mini Mental State Examination, RAVLT Rey Auditory Verbal Learning Test, VLS Victoria Longitudinal Study, WAIS-R Wechsler Adult Intelligence Scale-Revised.

Blood pressure represents cumulative blood pressure (per 100mmHg higher). Linear mixed models were used, adjusting for age, sex, current smoking, education, body mass index, presence of diabetes, treatment with antihypertensive medications at baseline, trajectory of systolic blood pressure change, and the corresponding baseline measure of cognitive function.

Negative relationships indicate that greater cumulative blood pressure exposures are associated with greater declines in cognitive scores over time.

# Table S10 cumulative exposure to blood pressure and change in cognition H70, excluding those with possible dementia

| Cognitive tests | Blood pressure | *n* | β (95% confidence interval) | *p* |
| --- | --- | --- | --- | --- |
| Immediate recall | Systolic | 401 | 0.06 (0.0005, 0.12) | 0.05 |
|  | Diastolic |  | 0.10 (-0.02, 0.22) | 0.10 |
| Delayed recall (from 2005) | Systolic | 281 | -0.12 (-0.32, 0.09) | 0.27 |
|  | Diastolic |  | 0.01 (-0.44, 0.45) | 0.97 |
| Semantic fluency | Systolic | 401 | -0.13 (-0.29, 0.02) | 0.08 |
|  | Diastolic |  | -0.29 (-0.58, 0.01) | 0.06 |
| General cognition (MMSE) | Systolic | 397 | 0.07 (0.02, 0.11) | 0.004 |
|  | Diastolic |  | 0.13 (0.04, 0.22) | 0.004 |

MMSE denotes Mini Mental State Examination.

Blood pressure represents cumulative blood pressure (per 100mmHg higher). Linear mixed models were used, adjusting for age, sex, current smoking, education, body mass index, presence of diabetes, treatment with antihypertensive medications, trajectory of systolic blood pressure change, and the corresponding baseline measure of cognitive function.

Negative relationships indicate that greater cumulative blood pressure exposures are associated with greater declines in cognitive scores over time.
